# Visfatin and Resistin Serum Levels in Normal-Weight and Obese Women With Polycystic Ovary Syndrome

**DOI:** 10.5812/ijem.15503

**Published:** 2014-07-01

**Authors:** Fatemeh Farshchian, Fahimeh Ramezani Tehrani, Houshang Amirrasouli, Hooman Rahimi Pour, Mehdi Hedayati, Faranak Kazerouni, Adeleh Soltani

**Affiliations:** 1 Medical Laboratory Technology Department, Shahid Beheshti University of Medical Sciences, Tehran, IR Iran; 2 Reproductive Endocrinology Research Center, Research Institute for Endocrine Sciences, Shahid Beheshti University of Medical Sciences, Tehran, IR Iran; 3 Faculty of Medicine, Shahid Beheshti University of Medical Sciences, Tehran, IR Iran; 4 Cellular and Molecular Research Center, Research Institute for Endocrine Sciences, Shahid Beheshti University of Medical Sciences, Tehran, IR Iran

**Keywords:** Polycystic Ovary Syndrome, Visfatin, Resistin, Obesity, Body Mass Index

## Abstract

**Background:**

Polycystic ovary syndrome (PCOS) is the most common endocrinopathy among women of reproductive age that is linked to insulin resistance and obesity. While studies have shown that plasma levels of resistin and visfatin increase with obesity, the association between PCOS and these markers has not been described well.

**Objectives:**

This case-control study aimed to compare the serum levels of visfatin and resistin in women with PCOS in comparison with the healthy controls matched for age and body mass index (BMI).

**Patients and Methods:**

A total of 80 women consisted of 40 women with PCOS and 40 matched eumenorrheic women without hyperandrogenism enrolled in the study. They were subcategorized into obese and normal-weight women according to their BMI. Serum visfatin and resistin levels were assessed using sandwich enzyme-linked Immunosorbent assay (ELISA).

**Results:**

Serum levels of resistin were higher among both obese and normal-weight women with PCOS in comparison with the controls (2.36 and 1.58 ng/mL in normal-weight women with PCOS and controls, respectively; and 2.10 and 1.91 ng/mL in obese women with PCOS and controls, respectively). Serum visfatin levels was higher in both obese women with PCOS and controls (3.46 and 3.49 ng/mL PCOS and control groups, respectively) in comparison with normal-weight women in both groups (3.16 and 3.15 in PCOS and control groups, respectively); however; there were no statistically significant differences in serum resistin and visfatin levels between PCOS and control groups (P > 0.05).

**Conclusions:**

While the expression of visfatin and resistin may be upregulated in women with PCOS, it is not translated at serum level.

## 1. Background

Polycystic ovary syndrome (PCOS) is a heterogeneous syndrome with a broad spectrum of reproductive, cosmetic, and metabolic manifestation including anovulation, hyperandrogenemia, polycystic ovaries, infertility, hirsutism, acne, insulin resistance (IR), dyslipidemia, and obesity (1-3). Thus, PCOS can lead to an increased risk of developing type 2 diabetes mellitus and cardiovascular disease (4). The etiology and pathogenesis of PCOS have remained unknown and it has various phenotypes including obese and insulin-resistant, obese and insulin-sensitive, normal-weight and insulin-resistant, and non-insulin-resistant types (5). The adverse effect of obesity with or without PCOS on reproduction and metabolic parameters has been studied thoroughly (6). Since obesity and IR are associated with PCOS in majority of patients, the molecules and hormones secreted by adipose tissue have been assumed to play a role in the pathogenesis of PCOS and therefore, were frequently investigated. It has been shown that adipose tissue plays an important role in the regulation of many physiological processes such as reproduction, immune response, and glucose and lipid metabolism through secretion of a variety of bioactive cytokines such as adipokines (7). Resistin is a small cysteine-rich protein secreted as a 94-amino acid polypeptide that was first named by Steppan et al. due to its IR property in mice (8). It is primarily secreted by mature white adipocytes and its circulating levels is increased in diet-induced or genetic forms of obesity and decreased by using the antidiabetic drug, eg, rosiglitazone. Human resistin, however, is mainly secreted by peripheral blood mononuclear cells (9) and its expression is predominantly localized in macrophages and stromal cells of adipose tissue rather than adipocytes (10, 11). Visfatin, previously known as pre-B-cell colony-enhancing factor (PBEF) (12), is a highly-conserved 52-kDa protein expressed in a variety of cell types and tissues including adipocytes, lymphocytes, bone marrow, liver, muscle, trophoblast, and fetal membranes. Visfatin was initially discovered and named by Fukuhara et al. who found that the mRNA level of a secreted protein was much more abundant in visceral fat than in subcutaneous fat (13). Visfatin binds to insulin receptor and exhibits insulin-mimetic actions, therefore, it stimulates glucose uptake in adipocytes and muscle cells and suppresses glucose release from hepatocytes (13, 14). Furthermore, it has been demonstrated that visfatin displays proinflammatory characteristics and modulated immune functions (15); however, there are contradictory data on the association between visfatin and obesity in women (16-21). Data regarding the levels of resistin and visfatin in patients with PCOS are still controversial; while some studies reported that visfatin levels is higher in women with PCOS (22-28), the other ones have not reported it (29-31), and although higher level of resistin in PCOS has been shown in one study (32), most studies reported no significant difference in resistin level between the PCOS the control groups (33-38).

## 2. Objectives

The aim of this study was to assess the association between serum levels of visfatin and resistin in obese and normal-weight women with PCOS and their age-BMI-matched healthy counterparts.

## 3. Patients and Methods

A group of 40 women diagnosed with PCOS based on National Institute of Health (NIH) diagnostic criteria for PCOS were recruited from the Reproductive Endocrinology Center data pool; forty age-BMI-matched healthy normo-ovulatory nonhirsute women referred to a gynecologic clinic for annual cervical cancer screening program were enrolled as controls. Participants had no history of oophorectomy or any other kind of ovarian surgery, hormonal treatment, and weight changes for at least three months before entering the study. The study protocol was approved by the Ethical Committee of the Research Institute for Endocrine Sciences and all participants signed an informed consent. A standard questionnaire including information about their age, age at menarche, reproductive history, and family history of hirsutism, oligomenorrhea, and infertility was completed for each subject. All participants underwent clinical examinations where body weight, height, and hirsutism score were assessed by trained staff. Height and weight were measured using standard apparatus with subjects in light clothes and without shoes. Weight was measured to the nearest 0.1 kg on a calibrated Beam scale. Height was measured to the nearest 0.5 cm with a measuring tape. Body mass index (BMI) was calculated as weight in kilograms divided by the square of height in meters (kg/m^2^). The blood samples were collected between the third and sixth day of their spontaneous or progesterone-induced menstrual cycles. Normal weight and obesity were defined as BMI between 18.5 and 24.9 kg/m^2^ and BMI ≥ 25.0 kg/m^2^, respectively. The control group was subcategorized into normal and obese according to their BMI: group A, normal-weight (n = 16); and group B, obese (n = 24) ‏ women. Subsequently, the case group was subcategorized: group C, normal-weight (n = 16); and group D, obese women (n = 24). PCOS was defined using the NIH criteria as the presence of anovulation and hyperandrogenism after exclusion of other known related disorders such as hyperprolactinemia, thyroid disorders, and nonclassical adrenal hyperplasia. Anovulation was considered as a history of eight or fewer menstrual cycles per year or a menstrual cycle length shorter than 21 or longer than 40 days. Hyperandrogenism was considered as the presence of hirsutism scores ≥ 8 using modified Ferriman-Gallwey (mFG) and/or free androgen index (FAI), androstenedione (A4), and/or dehydroepiandrosterone sulfate (DHEA-S) level above the upper 95^th^ percentile of the 40 healthy, nonhirsute, eumenorrheic controls. The FAI was calculated using the following formula: total testosterone (TT; nmol/L) × 100/sex hormone-binding globin (SHBG; nmol/L). Blood samples were obtained from the participants between 7:00 AM and 9:00 AM while sitting, after a 12-hour overnight fast. Samples were centrifuged within 30 to 45 minutes of collection and stored at -80℃.

### 3.1. Laboratory Procedures

Visfatin and resistin were measured by a sandwich enzyme-linked immunosorbent assay (ELISA) method (Human visfatin ELISA and Human Resistin, Cusabio Biotech Co Ltd, China). The lower detectable concentrations of visfatin and resistin were 0.156 ng/mL and 0.078 ng/mL, respectively. The intra-assay coefficients of variation were 6.8 and 7.3 for visfatin and resistin, respectively. DHEA-S, SHBG, and A4 were measured by ELISA (Human DHEA-s, Human SHBG, and Human A4 ELISA kit, Diagnostics Biochem Canada Inc., Ontario, Canada). All ELISA tests were performed using Sunrise ELISA reader (Tecan Co., Salzburg, Austria). The intra-assay and inter-assay coefficients of variation were respectively 5.6% and 6.6% for TT, 2.0% and 5.1% for DHEA-S, 1.2% and 5.7% for SHBG, and 2.2% and 3.5% for A4.

### 3.2. Statistical Analysis

Continuous variables were tested for normality using the one-sample Kolmogorov-Smirnov test; resistin, A4, DHEA-S, and FAI did not have normal distribution. The variables were expressed as mean and standard deviation or median and interquartile range. Distributions between groups were compared using the Kruskal-Wallis test, followed with Mann-Whitney U test with Bonferroni correction for pairwise comparison for resistin, A4, DHEA-S, and FAI and ANOVA test for visfatin. The categorical variables were compared using the Pearson’s test. Data analysis was performed using the SPSS 15.0 PC package (SPSS Inc., Chicago, IL, USA). Statistical significance was set at P < 0.05.

## 4. Results

Basic characteristics and the mean serum concentration of adipokines among women with PCOS and the controls are shown in Table 1 and 2, respectively. Serum resistin level was higher in women with PCOS‏ (group C, D) in comparison with the controls (group A, B), whereas no significant difference was seen between all groups. Serum visfatin levels was higher in obese women (group B, D) than normal-weight women (group A, C), whereas no significant difference existed between all groups. According to the Pearson test, there were no correlation between resistin and visfatin in the all study groups (Group A, r = - 0.08, p = 0.75; Group B, r = 0.21, p = 0.31; Group C, r = - 0.28, p = 0.28; and Group D, r = - 0.05, p = 0.78). (

**Table 1.** Characteristics of Study Participants According to Their Body Mass Index and Polycystic Ovary Syndrome Status^a,b,c^

Characteristics	Group A	Group B	Group C	Group D
**Age, y**	28.3 ± 4.8	34.0 ± 6.5	28.3 ± 5.1	34.2 ± 6.8
**BMI, kg/m** ^**2**^	22.4 ± 1.8	29.4 ± 3.5	22.5 ± 1.7	29.3 ± 3.4
**Hirsutism score** ^**d,e**^	0.2 ± 0.8	0.3 ± 0.8	9 ± 1.0	9.6 ± 2.1
**FAI** ^**d,e**^	1.3 (1.4-1.8)	1.4 (1.1-1.5)	3.0 (1.9-4.1)	3.3 (1.9-4.5)
**A4** ^**d,e**^ **, ng/mL**	1.5 (1.1-0.8)	1.6 (1.2-2.5)	2.6 (1.6-4.6)	3.4 (2.3-4.7)
**DHEA-S** ^**d,e**^ **, μg/dL**	126.4 (109.7-156.8)	175.1 (154.7-195.5)	182.3 (165.9-201.6)	204.3 (161.3-231.2)

^a^ Abbreviations: BMI, body mass index; DHEA-S, dehydroepiandrosterone sulfate; A4, androstenedione; and FAI, free androgen index.

^b^ Group A, non-PCOS with BMI < 25 kg/m^2^; Group B, non-PCOS with BMI ≥ 25 kg/m^2^; Group C, PCOS with BMI < 25 kg/m^2^; and Group D, PCOS with BMI ≥ 25 kg/m^2^.

^c^ Values are presented as mean ± SD or median (interquartile range).

^d^ Group A versus group C, p < 0.05.

^e^ Group B versus Group D, P < 0.05.

**Table 2.** The Serum Concentration of Visfatin and Resistin in Study Participants According to Their Body Mass Index and Polycystic Ovary Syndrome Status^a,b^

Adipokines	Group A	Group B	Group C	Group D	P Value
**Visfatin, ng/mL**	3.15 ± 1.06 (0.73-4.95)	3.49 ± 1.49 (0.63-6.16)	3.16 ± 0.93 (1.53-4.67)	3.46 ± 1.24 (1.73-6.66)	0.74^c^
**Resistin, ng/mL**	1.58 ± 2.57 (0.08-9.98)	1.91 ± 2.35 (0.21-10.03)	2.36 ± 3.29 (0.34-12.36)	2.10 ± 2.88 (0.08-11.65)	0.48^d^

^a^ Group A, non-PCOS with BMI < 25 kg/m^2^; Group B, non-PCOS with BMI ≥ 25 kg/m^2^; Group C, PCOS with BMI < 25 kg/m^2^; and Group D, PCOS with BMI ≥ 25 kg/m^2^ (abbreviations: PCOS, polycystic ovary syndrome; and BMI, body mass index).

^b^ Values are presented as mean ± SD (interquartile range).

^c^ Insignificant between all groups; comparison was made using ANOVA analysis.

^d^ Insignificant between all groups; comparison was made using Kruskal-Wallis test.

## 5. Discussion

The results of our study showed that although resistin and visfatin serum levels were higher in women with PCOS in comparison to controls, the differences were insignificant. This finding was consistent with several other studies (33-38). Seow et al. did not find any significant difference in either serum or follicular fluid resistin level between the PCOS and the control groups (37). In contrary to our study, Escobar-Morreale et al. showed that serum resistin levels were increased in overweight and obese women in comparison with normal-weight subjects, irrespective of having PCOS (33). Although it was reported that resistin mRNA levels were two-fold higher in adipocytes from patients with PCOS than in those from normal controls (37), adipocyte-produced resistin does not seem to play a key role in the body as adipocytes are not the major source of circulating resistin. Adipocyte resistin mRNA expression was reported to be significantly decreased after laparoscopic ovarian electrocautery in both obese and normal-weight women with PCOS (36). Moreover, no significant difference was observed in plasma resistin levels of patients with PCOS with regard to presence or absence of IR (38). On the other hand, Munir et al. found a 40% increase in mean serum resistin level in patients with PCOS and a positive correlation with BMI and testosterone level (32). In addition, resistin was observed to synergize with insulin to augment ovarian androgen production through enhancing 17-∝-hydroxylase mRNA expression and activity in ovarian theca cells. In a study on resistin promoter, no strong evidence was found for association of variation in resistin gene promoter with the phenotypes of PCOS (39). In contrary, the association of resistin gene polymorphism with BMI in women with PCOS suggests that resistin might be related to adiposity in PCOS (40). In addition, in a randomized placebo-controlled study, treatment with the insulin sensitizer, rosiglitazone, significantly reduced serum resistin levels in overweight women with PCOS, which implies that resistin contributes to insulin sensitivity improvement during treatment (41). It had been previously reported that the gene expression and circulating levels of visfatin were increased in women with PCOS in comparison with age-matched and BMI-matched controls (21-28); however, similar to our findings, several recently published studies did not find any difference in serum levels of visfatin between patients with PCOS and the control group (29-31). Guducu et al. reported that Serum visfatin levels were similar in patients with PCOS and controls. Visfatin levels were higher in normal-weight women with PCOS when compared with obese ones, but it did not reach statistical significance (29). Although the results of present study with age-BMI-matched case and control groups showed the higher serum visfatin level in obese than the normal-weight women irrespective of the presence of PCOS, no significant difference was observed in Visfatin levels between four subgroups.

It is interesting to know that visfatin levels in women with PCOS were significantly reduced after a three-month treatment with metformin and visfatin could differentiate between women with or without increased diabetogenic risk at a cutoff value of 19.24 ng/mL (25). In addition, such a treatment resulted in a significant decrease in BMI, homeostasis model assessment of IR, fasting insulin, and free testosterone, which suggests the possible involvement of visfatin in the pathophysiology of PCOS and its related complications. The results of our study showed that although resistin and visfatin serum levels were higher in patients with PCOS in comparison with controls, the difference was not statistically significant. It seems that while the expression of visfatin and resistin may be upregulated in women with PCOS, it is not translated at serum level. Thus Circulating serum visfatin and resistin levels may not reflect the events occurring at the adipocyte tissue levels. Further works are necessary to improve our understanding about the role of these two adipokines in reproductive functions that may act as a link between obesity and PCOS as well as their probable role in inflammatory process.
